# ACLY as a modulator of liver cell functions and its role in Metabolic Dysfunction-Associated Steatohepatitis

**DOI:** 10.1186/s12967-023-04431-w

**Published:** 2023-08-24

**Authors:** Paolo Convertini, Anna Santarsiero, Simona Todisco, Michele Gilio, Donatella Palazzo, Ilaria Pappalardo, Dominga Iacobazzi, Maria Frontuto, Vittoria Infantino

**Affiliations:** 1 Department of Science, University of Basilicata, Viale dell’Ateneo Lucano 10, 85100 Potenza, Italy; 2 Infectious Diseases Unit, San Carlo Hospital, Via Potito Petrone, 85100 Potenza, Italy; 3 Bristol Medical School, Translational Health Sciences, University of Bristol, Bristol, BS2 8HW UK

**Keywords:** ATP citrate lyase (ACLY), Hepatocytes, Metabolic Dysfunction-Associated Steatohepatitis (MASH), Oxidative stress, NF-kB

## Abstract

**Background:**

Non-alcoholic Fatty Liver Disease (NAFLD), now better known as Metabolic (Dysfunction)-Associated Fatty Liver Disease (MAFLD) and its progression to Nonalcoholic Steatohepatitis (NASH), more recently referred to as Metabolic (Dysfunction)-Associated Steatohepatitis (MASH) are the most common causes of liver failure and chronic liver damage. The new names emphasize the metabolic involvement both in relation to liver function and pathological features with extrahepatic manifestations. This study aims to explore the role of the immunometabolic enzyme ATP citrate lyase (ACLY), with a critical function in lipogenesis, carbohydrate metabolism, gene expression and inflammation.

**Methods:**

ACLY function was investigated in TNFα-triggered human hepatocytes and in PBMC-derived macrophages from MASH patients. Evaluation of expression levels was carried out by western blotting and/or RT-qPCR. In the presence or absence of ACLY inhibitors, ROS, lipid peroxidation and GSSG oxidative stress biomarkers were quantified. Chromatin immunoprecipitation (ChIP), transient transfections, immunocytochemistry, histone acetylation quantitation were used to investigate ACLY function in gene expression reprogramming. IL-6 and IL-1β were quantified by Lumit immunoassays.

**Results:**

Mechanistically, ACLY inhibition reverted lipid accumulation and oxidative damage while reduced secretion of inflammatory cytokines in TNFα-triggered human hepatocytes. These effects impacted not only on lipid metabolism but also on other crucial features of liver function such as redox status and production of inflammatory mediators. Moreover, ACLY mRNA levels together with those of malic enzyme 1 (ME1) increased in human PBMC-derived macrophages from MASH patients when compared to age-matched healthy controls. Remarkably, a combination of hydroxycitrate (HCA), the natural ACLY inhibitor, with red wine powder (RWP) significantly lowered ACLY and ME1 mRNA amount as well as IL-6 and IL-1β production in macrophages from subjects with MASH.

**Conclusion:**

Collectively, our findings for the first time highlight a broad spectrum of ACLY functions in liver as well as in the pathogenesis of MASH and its diagnostic and therapeutic potential value.

**Graphical Abstract:**

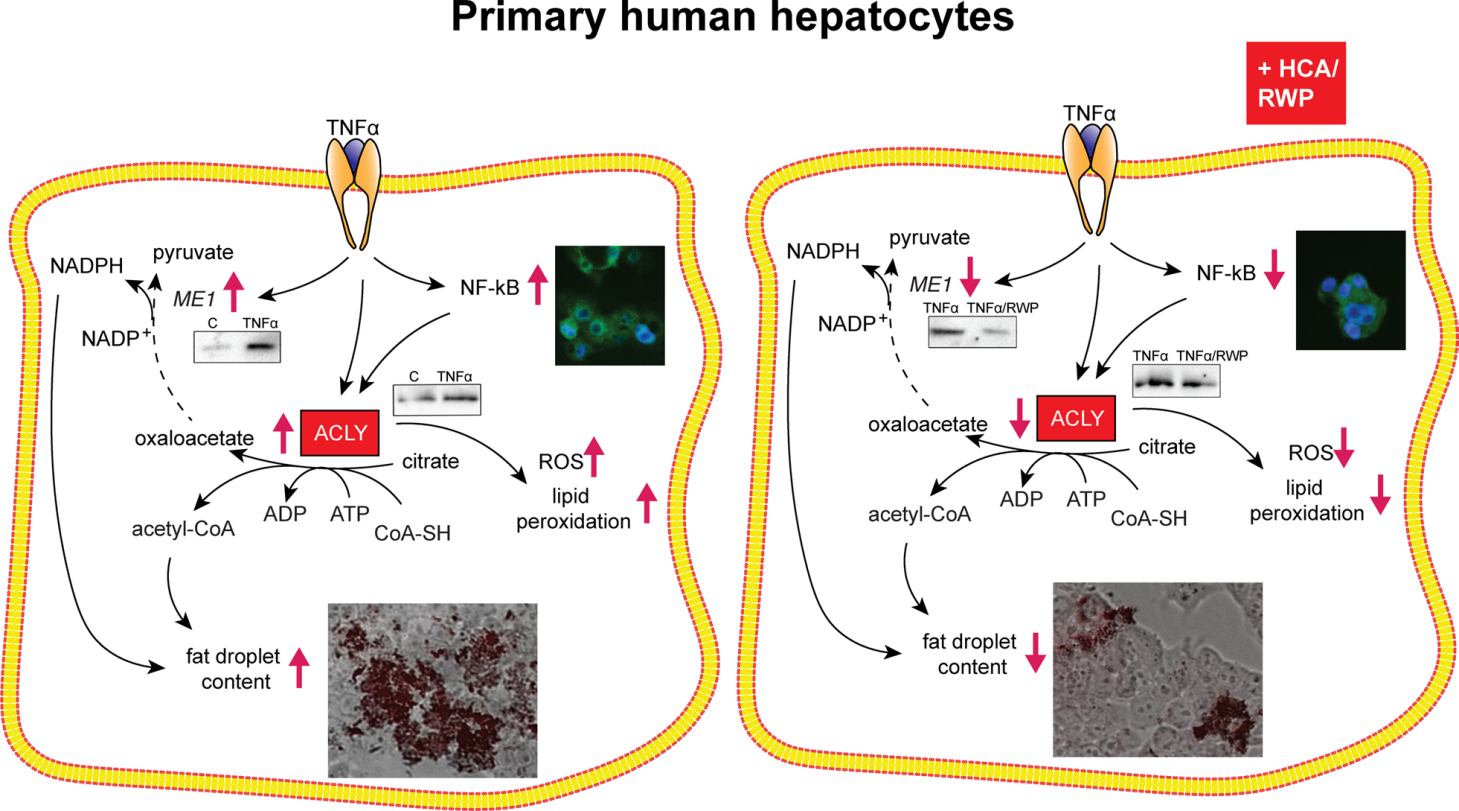

**Supplementary Information:**

The online version contains supplementary material available at 10.1186/s12967-023-04431-w.

## Background

The liver is a pivotal hub of energetic metabolism by connecting nutritional needs and metabolic outputs and integrating systemic metabolic signals. For this reason, metabolic dysfunctions strongly impact on liver function. A serious global health and economic burden affecting about a quarter of the world's adult population is represented by non-alcoholic fatty liver disease (NAFLD) [1]. In 2020, NAFLD has been changed to metabolic (dysfunction)-associated fatty liver disease (MAFLD), and this new term is now widely recognized by the international academic community [1]. The exclusion of different causes of chronic liver disease, including viral hepatitis or alcohol consumption, is important for MAFLD diagnosis [2]. By means of meta-analyses, the global prevalence of MAFLD has been estimated to be 37–39%. Furthermore, the incidence of MAFLD is constantly growing together with the crescent prevalence of metabolic syndrome, diabetes, cardiovascular disease as well as a large number of chronic metabolic diseases [3–5]. Sedentary behavior, reduced physical activity, and an excess caloric intake compared to expenditure have fostered the disease prevalence. Moreover, poor metabolic health in adults, even those of normal weight, is widespread in rich countries [6]. MAFLD ranges from a simple liver steatosis to a possible progression to steatohepatitis and cirrhosis [7]. Indeed, a considerable part of patients, affected by MAFLD, develop a more serious pathology such as nonalcoholic steatohepatitis (NASH), more recently referred to as metabolic (dysfunction)-associated steatohepatitis (MASH) characterized by hepatic inflammation and liver injury. More severely, MASH, can evolve into cirrhosis, and eventually hepatocellular carcinoma (HCC) [8].

A most recent approach, which has been looking at the liver as an immunological organ, might provide a better overview of the liver disease features. Indeed, a great number of innate immunocytes present hepatic localization. Among them, resident macrophages (Kupffer cells) as well as infiltrating monocyte-derived macrophages play key roles in MAFLD progression to MASH participating in both inflammation and hepatic homeostasis. A metabolic fatty acid-induced reprogramming of hepatic macrophages occurs in MAFLD development leading toward M1 phenotype [9].

Nearly a century ago, Otto Warburg traced a path towards understanding the pathogenic role of the metabolism by indicating metabolic dysregulations as hallmark of cancer cells [10]. Recently, the pivotal function of metabolism has reappeared not only in tumorigenesis but also in immune cell activation (innate and adaptive immunity). Countless studies support the idea that a metabolic reprogramming drives the phenotype of immune cells—hence, the meaning of immunometabolism—mainly by regulating gene expression [11]. Among the metabolic changes occurring during both macrophage and dendritic cell activation, a Krebs cycle rewiring has been observed [12]. In M1 macrophages, two breakpoints fragment the Krebs cycle: firstly at level of citrate, and a second one after succinate production. The last metabolite works by modulating Hypoxia-inducible factor 1-alpha (HIF-1α) stabilization and Interleukin-1β (IL-1β) production [13]. Citrate is exported via the mitochondrial citrate carrier (CIC) to the cytosol where the enzyme ATP citrate lyase (ACLY) cleaves it into oxaloacetate (OAA) and acetyl-Coenzyme A (acetyl-CoA). Interestingly, both CIC and ACLY are activated upon lipopolysaccharide (LPS) or Tumor Necrosis Factor-α (TNFα)/Interferon γ (IFNγ) stimulation. OAA, by conversion to malate (M) and then pyruvate, is a significant source of β-Nicotinamide adenine dinucleotide 2′-phosphate reduced (NADPH), electron donor for the production of superoxide anion (O2^**.**−^) and nitric oxide (NO) inflammatory mediators. Acetyl-CoA supplies carbon units for prostaglandin synthesis [14]. Moreover, ACLY-derived acetyl-CoA provides the acetyl groups involved in protein acetylation, among which histones and Nuclear Factor-kappa B (NF-kB) subunit p65, making ACLY a leading enzyme for gene expression reprogramming in activated macrophages [15, 16].

These new outcomes point out a more complex function of ACLY in cell physiology including redox state modulation of NADPH-dependent oxidant species, protein acetylation, gene expression and regulation of the inflammatory response. Remarkably, oxidative stress is a hallmark of MAFLD and one of the drivers toward MASH [17]. To this regard, lipid peroxidation has been extensively studied and lipid damage products are increased in MAFLD/MASH experimental model investigated [18].

However, until recently, the main function of the citrate export-induced ACLY was linked to the hepatic metabolism mainly due to its involvement in fatty acid biosynthesis. As a matter of fact, ACLY has been found overexpressed in HCC [19] and the role suggested in MASH is substantially referred to lipogenesis by directly affecting steatosis, dyslipidemia and hyperinsulinemia [20].

The purpose of our study is to thoroughly investigate the hepatic ACLY function taking into account the new findings in gene expression and redox modulation in order to better understand its role in liver and MASH development.

## Methods

### Cell culture and treatments

Primary Human Hepatocytes (HH, Lonza, Walkersville, MD, USA) were cultured in Hepatocyte Culture Medium (Lonza) according to the manufacturer’s protocol. HepG2 obtained from ATCC (Sigma-Aldrich, St Louis, MO, USA) were grown in CO_2_ (5%) incubator at 37 °C in high glucose Dulbecco’s modified Eagle’s medium (DMEM) GlutaMAX Supplement (Thermo Fisher Scientific, San Jose, CA, USA) after addition of 10% (v/v) fetal bovine serum, 100 U penicillin, and 100 μg/mL streptomycin. Human cell lines were tested periodically for mycoplasma by using MycoAlert PLUS detection kit (Lonza). Cells were treated with 500 µM hydroxycitrate (HCA, Sigma-Aldrich) or 200 µg/mL red wine powder (RWP) obtained as previously described from the Italian red wine *Aglianico del Vulture* [21]. After 1 h of treatment with HCA or RWP, cells were stimulated with 5 ng/mL of TNFα (Sigma-Aldrich) for up to 24 h. Where indicated, cells were treated also with 20 μM IKK inhibitor VII (IKK 16, Sigma-Aldrich), 5 mM sodium acetate (Ac, Sigma-Aldrich) or 5 mM sodium malate (M, Sigma-Aldrich) alone or in combination with 500 μM NADPH (Sigma-Aldrich).

### Lipid accumulation assay

Oil Red O staining was used for the detection of neutral lipids. Stock solution was obtained by dissolving 0.06 g Oil Red O (Sigma-Aldrich) in 20 mL isopropanol 100% and left undisturbed at room temperature for 20 min. Oil Red O working solution was prepared by diluting 3 parts of stock solution with 2 parts of distilled water. Working solution, stable for 2 h, was filtered immediately before use. For qualitative analysis, HH were seeded (1 × 10^5^ cells/well) in a 24-well plate and, the day after, treated with TNFα 5 ng/mL in the presence or not of 500 µM HCA or 200 µg/mL RWP. Twenty-four hours later, HH were washed twice with phosphate buffered saline (PBS) and fixed with 4% paraformaldehyde in PBS for 30 min at room temperature. Following two washing with distilled water, isopropanol 60% was added for 5 min. The cells were incubated with Oil Red O working solution for 20 min at room temperature. Then, HH were thoroughly washed three times with distilled water to remove the unbound staining solution and observed under fluorescence microscopy FLoid Cell™ Imaging Station (Thermo Fisher Scientific).

For quantitative analysis, after the incubation with Oil Red O working solution, the staining was extracted in isopropanol 100%, and lipid accumulation was quantified at 490 nm by using a microplate reader (GloMax® Discover Microplate Reader (Promega). The results are shown as percentage of the control (untreated cells), defined as 100% of neutral fats accumulation.

### Collection of human MASH samples

Anonymized human whole venous blood samples from 8 patients with MASH were obtained from the Department of Infectious Disease of San Carlo Hospital in Potenza, Italy. The same number of healthy controls were enrolled among hospital staff and volunteers. Samples were collected from May 2022 to March 2023. The diagnosis of Hepatic steatosis was based on Hepatic Steatosis Index (HSI). This score is founded on the use of ultrasound in order to show hepatic steatosis and on the presence of any of the three conditions of overweight/obesity, diabetes, and increased transaminases [22]. Experienced ultrasound physician blinded to the study further classified it as [1] mild steatosis (presence of diffuse echogenic enhancement or hepatorenal contrast) or [2] moderate or severe steatosis (both bright echogenic and hepatorenal contrast enhancement visible or ultrasound beam attenuation observed). All subjects provided written, informed consent, approving, and authorizing the use of their material for research purposes. Research was carried out in accordance with the Declaration of Helsinki and in agreement with local Italian Committee on Human Research’s approved procedures (REF. TS/CEUR 20200034750—15 September 2020).

### Isolation of PBMCs from whole blood and differentiation of human monocytes

Venous blood was collected into K2 EDTA-coated BD vacutainer tubes (Becton, Dickinson and Company, Franklin Lakes, NJ, USA). Peripheral blood mononuclear cells (PBMCs) were isolated using Histopaque-1077 (Sigma-Aldrich) density gradient centrifugation and human monocytes were obtained from PBMCs following incubation with CD14 antibody conjugated to magnetic beads (MACS®, Miltenyi Biotec GmbH, Bergisch Gladbach, Germany) as previously described [23]. The CD14^+^ monocytes were differentiated to macrophages by culturing in Roswell Park Memorial Institute (RPMI) 1640 medium (Thermo Fisher Scientific) supplemented with 10% fetal bovine serum, 2 mM l-glutamine, 100 U/mL penicillin, 100 μg/mL streptomycin, and 100 ng/mL recombinant human Macrophage Colony Stimulating Factor (M-CSF, Cell Guidance Systems, St. Louis, MO, USA) for 3 days at 37 °C in a humidified atmosphere of 5% CO_2_.

### Quantitative real-time PCR (RT-qPCR)

Total RNA was extracted from 2 × 10^6^ cells by RNeasy Plus Mini Kit (Qiagen, Hilden, Germany) as per manufacturer’s instructions. Complementary DNA was synthesized from 1 μg of RNA by iScript™ cDNA Synthesis Kit (Bio-Rad Laboratories, Hercules, CA, USA) (5 min at 25 °C, 20 min at 46 °C and 1 min at 95 °C) as per manufacturer’s guidelines. Real-time PCR experiments were performed in triplicate on the 7500 Fast Real-Time PCR System (Thermo Fisher Scientific) with human ACLY (Hs00982738, RefSeq NM_001096.2), ME1 (Hs00159110, RefSeq NM_002395.5) and β-actin (Hs01060665, RefSeq NM_001101.3) TaqMan Gene Expression Assays (Thermo Fisher Scientific). Data were analyzed according to the ΔΔCt method, as previously reported [24]. In detail β-actin was used as endogenous reference gene to obtain ΔCt value by subtracting the β-actin Ct value from the target gene (ACLY or ME1) Ct value, where Ct refers to the threshold cycle. The fold changes in the ACLY or ME1 expression in treated cells or patients relative to unstimulated cells or healthy controls were estimated by ΔΔCt method [25] by using the ΔCt mean values for calculations, unless otherwise specified. Fold changes were determined as 2^−ΔΔCt^.

### Western blot analysis

The lysate obtained from 1 × 10^6^ cells as previously reported [26] was subjected to a Bradford assay (Pierce™ Coomassie (Bradford) Protein Assay Kit (Thermo Fisher Scientific) to determine protein concentration using Bovine Serum Albumin as standard. Thirty micrograms of proteins were resolved by 8–12% sodium dodecyl sulfate-polyacrylamide gel electrophoresis (SDS-PAGE) and transferred onto nitrocellulose membranes. The membranes were blocked with a tris-buffered saline solution containing 5% non-fat dry milk and 0.5% Tween for 1 h at room temperature. Subsequently, membranes were probed overnight at 4 °C with anti-ACLY (ab157098, Abcam, Cambridge, MA), anti-ME1 (ab97445, Abcam), anti-NF-κB/p65 (ab16502, Abcam) or anti-β-actin (ab8227, Abcam) primary antibodies. The membranes were then incubated with horseradish peroxidase (HRP) conjugated goat anti-rabbit secondary antibody (Santa Cruz Biotechnology, Santa Cruz, CA, USA) for 1 h at room temperature. The immunoreactions were detected by WesternBright™ ECL (Advansta, Menlo Park, CA, USA) at Chemidoc™ XRS detection system (Bio-Rad Laboratories). Image Lab Software (Bio-Rad Laboratories) was used for image acquisition and densitometric analysis.

### ACLY activity

HH (1 × 10^7^) were treated for 3 h with 5 ng/mL TNFα, collected, and washed twice in ice-cold PBS. ACLY activity was assessed as previously reported [23] on the cell lysate obtained after three freeze-melt cycles (− 80 °C for 8 min/40 °C for 4 min) in ice-cold 0.1% Nonidet P40 (NP40)/PBS solution. The specific ACLY activity was normalized to the protein concentration and expressed as a percentage of the control (set at 100%).

### ROS detection

To detect reactive oxygen species (ROS) levels, HH (5 × 10^5^) were triggered by 5 ng/mL TNFα for 24 h in the presence or absence of 500 µM HCA or 200 µg/mL RWP. Where indicated, cells were co-treated with 5 mM Ac or 5 mM M alone or in combination with 500 μM NADPH. After 24 h, ROS concentrations were measured by using Di(Acetoxymethyl Ester) (6-Carboxy-2′,7′-Dichlorodihydrofluorescein Diacetate) (Thermo Fisher Scientific) as described in [27].

### Lipid peroxidation

Lipid peroxidation was monitored by the Lipid Peroxidation (MDA) Colorimetric/Fluorometric Assay Kit (BioVision, Milpitas, CA, USA) as previously reported [28]. HH (1 × 10^7^) were triggered by TNFα in the presence or not of HCA or RWP. In some experiments, cells were also treated with Ac or M alone or in combination with NADPH. After 24 h treatment, cells were collected and homogenized on ice in 300 μL of Malondialdehyde (MDA) Lysis Buffer. The supernatants, collected by centrifugation, were placed in microcentrifuge tubes to which Thiobarbituric Acid (TBA) reagent was added. After the incubation at 95 °C for 1 h and cooling at room temperature in an ice bath for 10 min, 200 μL of each sample, run in triplicate, was added to a 96-well plate. The MDA-TBA adduct was quantified fluorometrically (Ex/Em = 532/553 nm) by using GloMax® Discover Microplate Reader (Promega). The MDA amount was further calculated according to the kit manufacturer’s protocol.

### Glutathione detection

The levels of oxidized glutathione (GSSG) in human hepatocytes were evaluated by Glutathione Fluorometric Assay Kit (BioVision) at the end of 24 h treatment with TNFα in the presence or not of HCA or RWP. Cells (4 × 10^6^) were collected and homogenized on ice with 100 μL of ice-cold Glutathione Assay Buffer. Sixty μL of each homogenate was added in a prechilled tube containing perchloric acid (PCA) and vortexed for a few seconds to obtain a uniform emulsion. Supernatants were collected by centrifugation and neutralized by adding ice-cold 6N Potassium hydroxide (KOH) to precipitate PCA. To 10 µL of neutralized samples transferred into a 96-well plate, Assay Buffer and GSH Quencher were added. Following 10 min incubation at room temperature, Reducing Agent Mix was added to destroy the excess GSH Quencher and convert GSSG to GSH (reduced glutathione). After 40 min of incubation with OPA Probe, the fluorescence of each well was measured with GloMax® Discover Microplate Reader (Promega) by the mean of Fluorescence Excitation Modules and Emission Filters for UV (365nm_Ex_/415–445nm_Em_). The GSSG levels were further calculated according to the kit manufacturer's protocol and expressed as percentage of control.

### Transient transfection

For monitoring the promoter activity of ACLY gene, transient transfection was achieved using pGL3 basic-LUC vector (Promega) containing the − 3116/ − 20 bp region of the ACLY gene promoter (called “3000” and including the binding site for NF-κB) or a deletion fragment of this region (without NF-κB binding site, indicated as “1000”), and with 10 ng of pRL-CMV (Promega) to normalize the extent of transfection. The transfection was performed when the cells became 50% to 70% confluent, using Lipofectamine 3000 (Thermo Fisher Scientific) at a ratio of ~ 3.5 μg:5 μL/well (DNA: Lipofectamine). Briefly, HepG2 cells were seeded into a 24-well plate (5 × 10^4^ cells/well). The day after, the DNA-lipofectamine complex was made by mixing 25 μL of Opti-MEM Reduced Serum Medium containing 0.75 μL of lipofectamine 3000 and 25 μL of the medium containing 0.5 μg of DNA. The DNA-lipofectamine mixture was incubated for 15 min at room temperature. Finally, 50 μL of the DNA-lipofectamine mixture was added to the well containing the cells. Twenty-four hours after transfection, HepG2 cells were triggered by TNFα in the presence or absence of HCA. The next day, cells were lysed and assayed for LUC activity by using the Dual-Luciferase® Reporter Assay System (Promega), according to the manufacturer’s protocol. Luminescence was measured on GloMax® Discover Microplate Reader from Promega.

### ChIP-qPCR

For chromatin immunoprecipitation (ChIP) experiments, HH cells (5 × 10^6^) were treated with TNFα in the presence or absence of HCA for 3 h, then fixed by 1% formaldehyde at 37 °C for 10 min. Subsequently, HH cells were lysed in a 0.1% NP40/PBS buffer supplemented with protease inhibitors, put on ice for 10 min, and centrifuged at 1000×*g* for 2 min at 4 °C. Pellet resuspension in a 0.1% NP40/PBS buffer was followed by sonication at 70% power at cycle 9 for 10 min in a 0.1% SDS lysis buffer to generate cellular chromatin fragments of about 400 bp. Chromatin immunoprecipitation was performed overnight at 4 °C on a rocking platform using A/G PLUS agarose beads (Santa Cruz) with anti-NF-κB/p65 antibody (ab16502, Abcam). The following day, all samples, including the input (total chromatin extract) and mock (immunoprecipitation without the antibody), were recovered. The protein/DNA complexes were washed with PBS, then treated with RNase and Protease K. DNA was purified by means of PureLink™ PCR Purification Kit (Thermo Fisher Scientific) and analyzed by qPCR using the following primers: For-ACLY 5′-CTTTCCAAAGTTGGGTCTTGTG-3′ and Rev-ACLY 5′-CCTCAGCAATTCAGACTCCTT-3′. Each qPCR reaction mixture contained SYBR™ Green PCR Master Mix (Thermo Fisher Scientific), 20-pmol forward and reverse oligonucleotides, 200 ng DNA, and nuclease-free water was run on a 7500 Fast Real-Time PCR System (Thermo Fisher Scientific). Melting curve analysis was performed to verify the specificity of designed primers.

### Immunocytochemistry

HH (5 × 10^5^), following 3 h treatment with 5 ng/mL TNFα in the presence or absence of 200 µg/mL RWP, were subjected to immunocytochemistry experiments by using anti-NF-κB/p65 (ab7970, Abcam) as primary antibody and Alexa Fluor 488 (Thermo Fisher Scientific) as a secondary antibody according to the protocol described in [21]. Nuclear localization was calculated as % Nuclear according to the equation reported in [29] : % *Nuclear* =  [*Total Nuclear Intensity*/(*Total Cytoplasmic Intensity* + *Total Nuclear Intensity*)] × 100. Total fluorescence intensities were determined by using ImageJ software (https://imagej.nih.gov/ij/index.html, NIH, accessed on 12 July 2023).

### Global H3 and H4 acetylation assay

The effects of TNFα alone or in combination with HCA on H3 and H4 histone acetylation were evaluated with the EpiQuik™ Global Histone H3 Acetylation Assay Kit and EpiQuik™ Global Histone H4 Acetylation Assay Kit (Epigentek, Farmingdale, NY, USA). Following the manufacturer’s instructions, histones were extracted from HH that had been treated for 24 h with 5 ng/mL TNFα alone or combined with 500 μM HCA. The histones were spotted onto the wells, and then an antibody specific for acetylated histone H3 or H4 was added. After washing the wells, HRP-conjugated secondary antibody was added, followed by the detection reagent, and absorbance was measured with GloMax® Discover Microplate Reader at 450 nm. Percent acetylation was calculated as OD (treated sample − blank)/OD (untreated control − blank) × 100%.

### Quantification of cytokines

Twenty-four hours after stimulation with TNFα in the presence or not of HCA and/or RWP, cell-free supernatants were collected and assayed for the concentration of interleukins 1β and 6 by the mean of Lumit™ IL-6 (Human) Immunoassay and Lumit™ IL-1β (Human) Immunoassay (Promega) following the manufacturer’s recommendations.

### Statistical analysis

The statistical analysis of data was carried out employing the statistic tools implemented in the GraphPad Prism software (La Jolla, CA, USA). All results are presented as mean ± standard deviation (SD) from at least three independent experiments, each run in triplicate. For pairwise comparisons, Student’s t-test test was performed. Comparisons of more than two groups were made using one-way ANOVA followed by Tukey’s post hoc test. The statistical method used for each experiment is detailed in the figure legends. The asterisks in the figures denote statistical significance (*p < 0.05; **p < 0.01; and ***p < 0.001). When Tukey’s test was performed, different letters indicated significant differences between treatments at p < 0.05.

## Results

### TNFα activates ACLY and Malic Enzyme 1 in primary human hepatocytes

Circulating TNFα shows increased levels in animal model with MASH [30] as well as in patients with MAFLD compared to control subjects, along with different disease markers. Moreover, a high amount of circulating TNFα has been linked to MAFLD severity [31] while TNFα signaling inhibition lowers liver steatosis and hepatocellular injury in MAFLD mouse model. It is well known that treatment with TNFα promotes inflammation, lipid accumulation (a hallmark of MASH [32])—including fatty acid and cholesterol—and injury in hepatic cells [33–35]. Therefore, we used TNFα to trigger liver damage pathways and in turn to explore ACLY function.

**Fig. 1:**
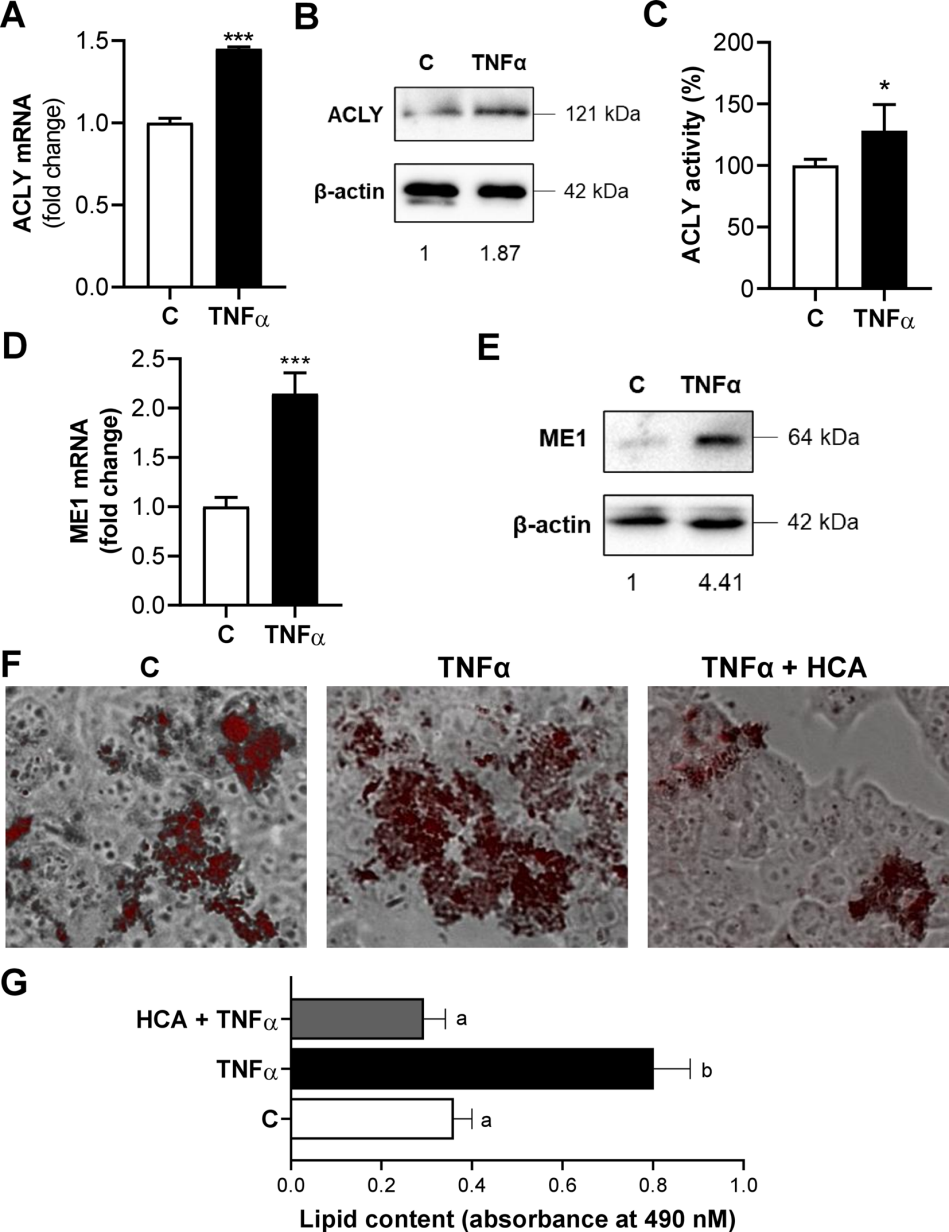
ACLY and ME1 are activated by TNFα in primary human hepatocytes. Primary human hepatocytes (HH) were treated with 5 ng/mL TNFα alone (TNFα) or combined with 500 μM HCA (TNFα + HCA). Unstimulated cells (C) were used as a negative control. Real time PCR experiments were performed to evaluate ACLY (**A**) and ME1 (**D**) mRNA levels. Data are representative of 3 independent experiments and are presented as mean ± SD (error bars). Western blot analyses revealed ACLY (**B**) and ME1 (**E**) protein content. Immunoblot data are representative of at least 3 independent experiments. Values obtained after normalization to β-actin are reported under western blot images. Protein expression levels in untreated HH (C) were taken as 1. **C** ACLY activity in human hepatocytes. **F** Representative photomicrographs of intracellular lipids staining. Photographs typical of those taken three separate experiments are shown. **G** Quantitative assessment of Oil Red O staining. Data are shown as mean ± SD (error bars) and derived from 3 experiments. In **A**, **C**, **D** differences were significant according to Student’s t-test (*p < 0.05 and ***p < 0.001). Statistical significance of difference was evaluated in **G** by the mean of one-way ANOVA followed by Tukey’s multiple comparison test and different letters indicate significant differences between treatments at p < 0.05

First of all, we tested the effect of TNFα on the expression levels of ACLY, the key metabolic enzyme in fatty acid metabolism as well as in oxidative stress and inflammation, both peculiar features of MASH, in primary human hepatocytes. Following 3 h stimulation with TNFα, we observed increases in ACLY mRNA (1.45 ± 0.01-fold change, Fig. 1A), protein levels (1.87, Fig. 1B) and enzymatic activity (128.35 ± 21.06, Fig. 1C) compared to untreated cells.

In the same condition, we recorded a double amount of cytosolic ME1 mRNA (Fig. 1D) and a 4.4-increment of protein (Fig. 1E) in TNFα-triggered HH cells than control. ME1 participates in the recycle of ACLY-produced OAA from cytosol to mitochondria with concomitant formation of NADPH reducing equivalents, essential to both fatty acid and inflammatory mediator biosynthesis.

In light of these results, we investigated the effect of HCA, a potent competitive inhibitor of ACLY, on lipid accumulation. Lipid content was assessed by using Oil Red O staining in HH treated with TNFα, individually or in combination with HCA. The concentration of HCA used was the same used in [36], that did not affect HH cell viability measured as described in Additional file 1  (Additional file 2: Fig. S1A). In comparison to the control, treatment with TNFα increased 2.2-fold the lipid accumulation (p < 0.001) (Fig. 1F, G). Instead, the fat droplet content was radically reduced to levels lower than the control ones by the combination treatment of TNFα and HCA (Fig. 1F, G). These findings suggest that ACLY and ME1 are overexpressed and the specific ACLY inhibition by HCA reduces fat droplet accumulation in TNFα-triggered HH.

### Effect of ACLY inhibition on oxidative stress biomarkers

Increased oxidative stress plays a central role in MAFLD pathogenesis and in its progression to MASH [17]. Indeed, MASH development is characterized by an increase in lipid peroxidation and ROS levels, together with a decrease in antioxidant defenses, GSH amount, and an increment in GSSG levels [37].

**Fig. 2:**
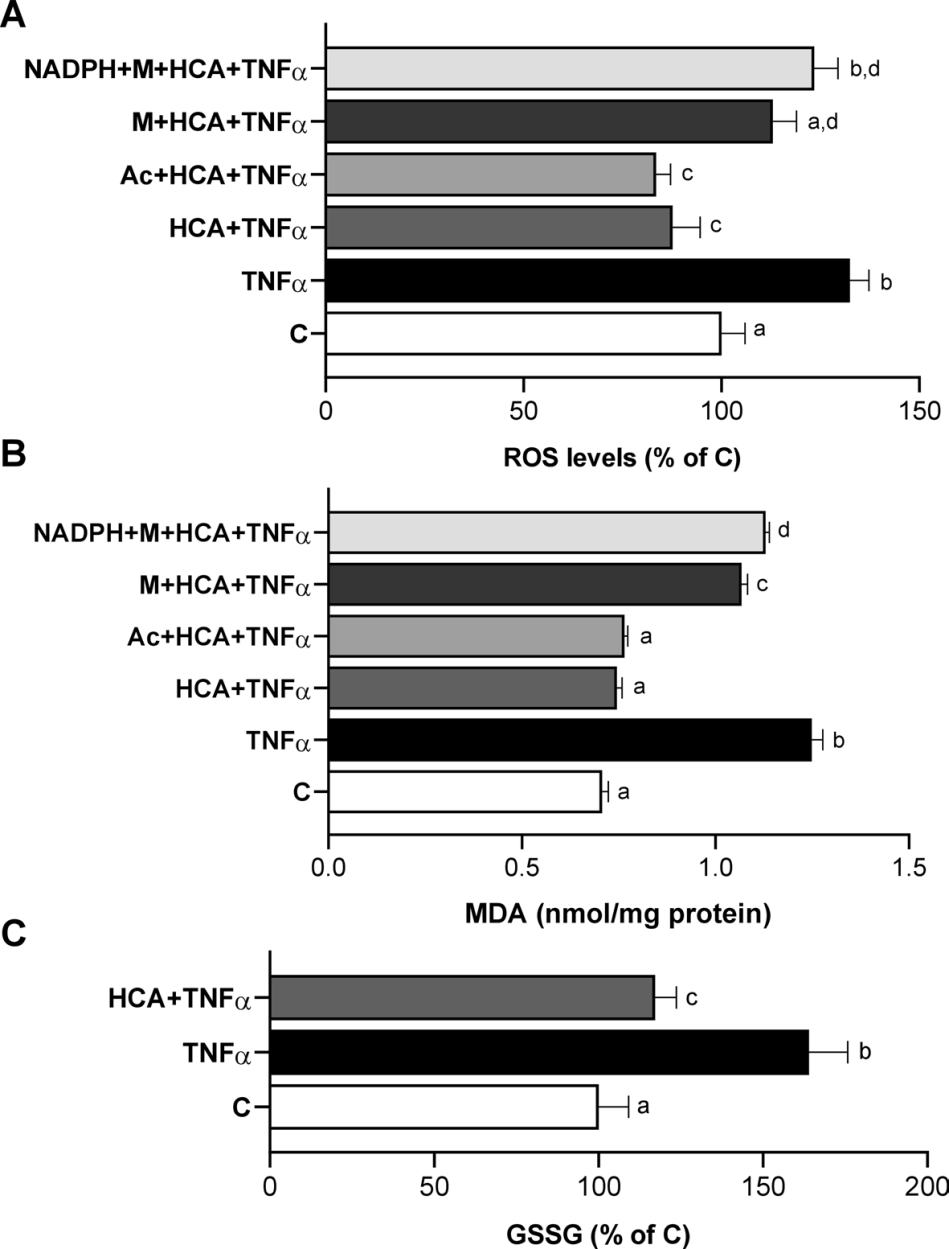
Effect of ACLY inhibition by HCA on ROS, lipid peroxidation and GSSG. HH cells were treated with 5 ng/mL TNFα in the absence (TNFα) or in the presence of 500 μM HCA (TNFα + HCA). Unstimulated cells (C) were used as a negative control. In **A**, **B** TNFα + HCA cells were cotreated with 5 mM sodium acetate (Ac) or 5 mM sodium malate (M) alone or in combination with 500 μM NADPH for 24 h and ROS (**A**) and MDA (**B**) levels were measured. In **C** GSSG levels are shown. Mean values ± SD of three independent experiments with at least three replicates in each are shown. In **A** and **C** values are expressed as the percentage of unstimulated cells (C, set at 100%). Statistical significance of differences was evaluated by using one-way ANOVA followed by Tukey’s multiple comparison test. Different letters indicate significant differences between treatments at p < 0.05

Although some of the mechanisms responsible for altered redox status are known, it cannot be ruled out that other processes, currently unknown, may contribute to it. In keeping the activation of ACLY (Fig. 1A–C) and its involvement, as part of the citrate pathway, in oxygen radical production [14] we assessed ROS levels in TNFα-triggered HH in the presence of HCA. As expected, TNFα caused an enhanced and significant release of ROS (mean ± SD = 132.5 ± 4.8, Fig. 2A) in comparison to control (mean ± SD = 100 ± 5.9). HCA, on the other hand, reduced by 35% the levels of ROS compared to cells treated only with TNFα (Fig. 2A). The addiction of exogenous acetate, which can provide acetyl-CoA (a product of ACLY activity) did not revert this decrease of ROS levels (Fig. 2A). On the contrary, the addiction of exogenous sodium malate alone or combined with NADPH, two players of the OAA recycle and produced downstream of ACLY, reverted the effect of ACLY inhibition. In fact, the malate alone led to a significant increase (p < 0.001) of about 30% in ROS levels compared to cells treated with TNFα and HCA and even a more marked increase, greater than 40%, when NADPH was also present (TNFα + HCA = 87.7 ± 6.9; TNFα + HCA + M + NADPH = 123.5 ± 6.0, Fig. 2A). Among the different consequences of oxidative stress with increase of ROS levels is the lipid peroxidation of membrane phospholipids. The peroxidation of arachidonic acid produces different peroxides and finally toxic aldehydes as well as MDA. The latter reacts with amino groups of proteins and DNA by forming adducts that can alter the function of these macromolecules in cells [38]. Therefore, we evaluated the outcome of ACLY inhibition by HCA on MDA levels. A significant increase of about 80% in lipid peroxidation was observed in TNFα-triggered HH compared to untreated cells while the addition of HCA drastically lowered the levels bringing them back to values similar to the control ones (Fig. 2B). As observed for ROS, the addition of sodium acetate had no effect; on the contrary, sodium malate alone or plus NADPH led to strong increase (of about 45% and 52%, respectively) in MDA levels compared to cells treated with TNFα and HCA (Fig. 2B). As expected, similar results of ROS and MDA reductions were obtained when ACLY was silenced - as described in Additional file 1 - by a specific siRNA targeting human ACLY (siACLY) (Additional file 2: Fig. S2). In detail, we recorded significant decreases (p < 0.001) of 30 and 25% in the content of ROS and MDA, respectively, in the presence of siACLY compared to cells treated only with TNFα (Additional file 2: Fig. S2).

GSSG levels increased by 65% in HH cells treated with the pro-inflammatory cytokine TNFα compared to those of the untreated cells, highlighting the oxidative stress condition. It is interesting to note that GSSG levels were significantly reduced by about 30% in HH cells pretreated with HCA and exposed to TNFα for 24 h (Fig. 2C). These findings confirm the alteration in glutathione homeostasis, observed in MASH where the hepatic accumulation of GSSG has been related to the regulation of oxidative stress effects, suggesting a synergistic effect of TNFα and GSSG levels in the induction of hepatocyte damage [37].

These outcomes, running parallel to ACLY and ME1 activation, indicate that the blocking of ACLY, through inhibition or gene silencing, may improve the redox status of hepatocytes.

### RWP inhibits ACLY and ME1 and improves the redox status

**Fig. 3:**
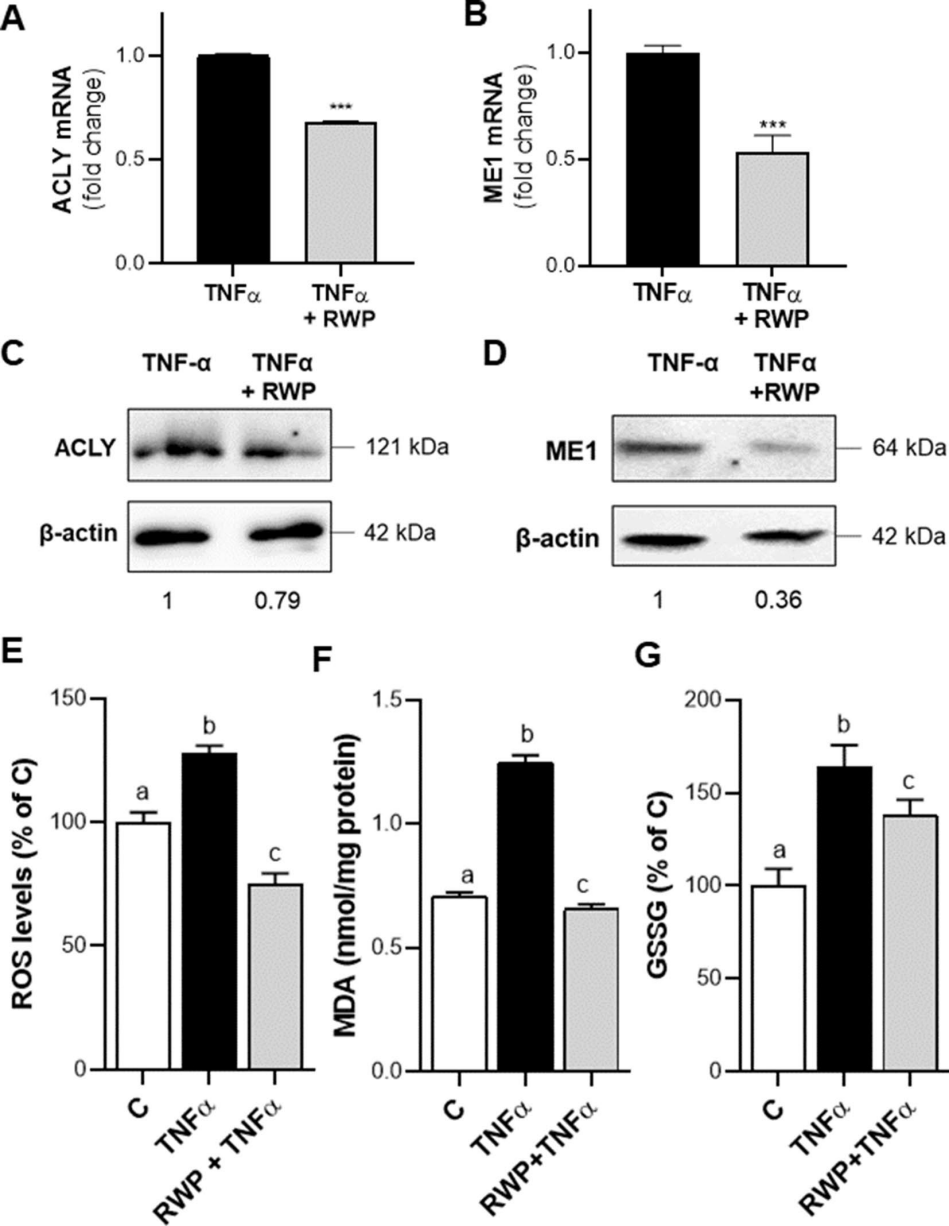
Effect of RWP on ACLY and ME1 expression and on oxidative stress. HH were triggered by 5 ng/mL TNFα in the absence (TNFα) or in the presence of 200 μg/mL RWP (TNFα + RWP). Via real time PCR analyses ACLY (**A**) and ME1 (**B**) mRNA fold changes were quantified. Western blot analyses were performed to measure ACLY (**C**) and ME1 (**D**) protein levels. Values obtained after normalization to β-actin are reported under western blot images. Protein expression levels in untreated HH (C) were taken as 1, and other samples were expressed in proportion to the control. Immunoblot data are representative of at least 3 independent experiments. In **E**–**G** ROS, lipid peroxidation and GSSG were evaluated following 24 h treatment with TNFα. Unstimulated cells (C) were used as a negative control. In **E** and **G** values are expressed as the percentage of unstimulated cells (C, set at 100%). In **A**, **B**, **E**–**G** mean values ± SD of three independent experiments with at least three replicates in each are shown. In **A**, **B** differences were significant according to Student’s t-test (***p < 0.001). In **E**–**G** statistical analysis was performed by using one-way ANOVA followed by Tukey’s multiple comparison test. Distinct letters indicate significant differences between treatments at p < 0.05

To further confirm the central role of ACLY in liver function, we tested the effect of ACLY inhibition by RWP, deriving from *Aglianico del Vulture* red wine, in our in vitro model. Notably, RWP restores LPS-triggered macrophage homeostasis via inhibition of both NF-kB and the citrate pathway [21]. After verifying that RWP did not affect HH viability (Additional file 2: Fig. S1B), we treated TNFα-activated HH with 200 μg/mL RWP, and RT-qPCR experiments evidenced a 35% reduction in ACLY mRNA (Fig. 3A) and a halving of ME1 gene expression levels (Fig. 3B). A similar trend of more marked reduction effect of ME1 than of ACLY by RWP was observed on protein expression: ACLY was reduced by about 20% (Fig. 3C) while ME1 by ~ 75% (Fig. 3D). Similarly to HCA, RWP drastically reduced lipid content in TNFα-activated HH restoring levels to values comparable to untreated cells (Additional file 2: Fig. S3A, B). Our analysis showed that RWP was able, like HCA, to improve the cellular redox status leading to a decrease in the levels of ROS (Fig. 3E), MDA (Fig. 3F) and GSSG (Fig. 3G) with respect to cells treated with TNFα alone. Once again, the modulation of ROS and lipid peroxidation occurred through ACLY since the addition of M, alone or plus NADPH, led to increased levels of both ROS and MDA (Additional file 2: Fig. S3C, D). Conversely, sodium acetate, which supplies acetyl units, i.e. the other metabolite produced by ACLY, had no effect (Additional file 2: Fig. S3C, D). Thus, the significant role played by RWP in restoring liver homeostasis is mainly dependent on ACLY.

### Crosstalk between NF-kB and ACLY in TNFα induced hepatocytes

**Fig. 4:**
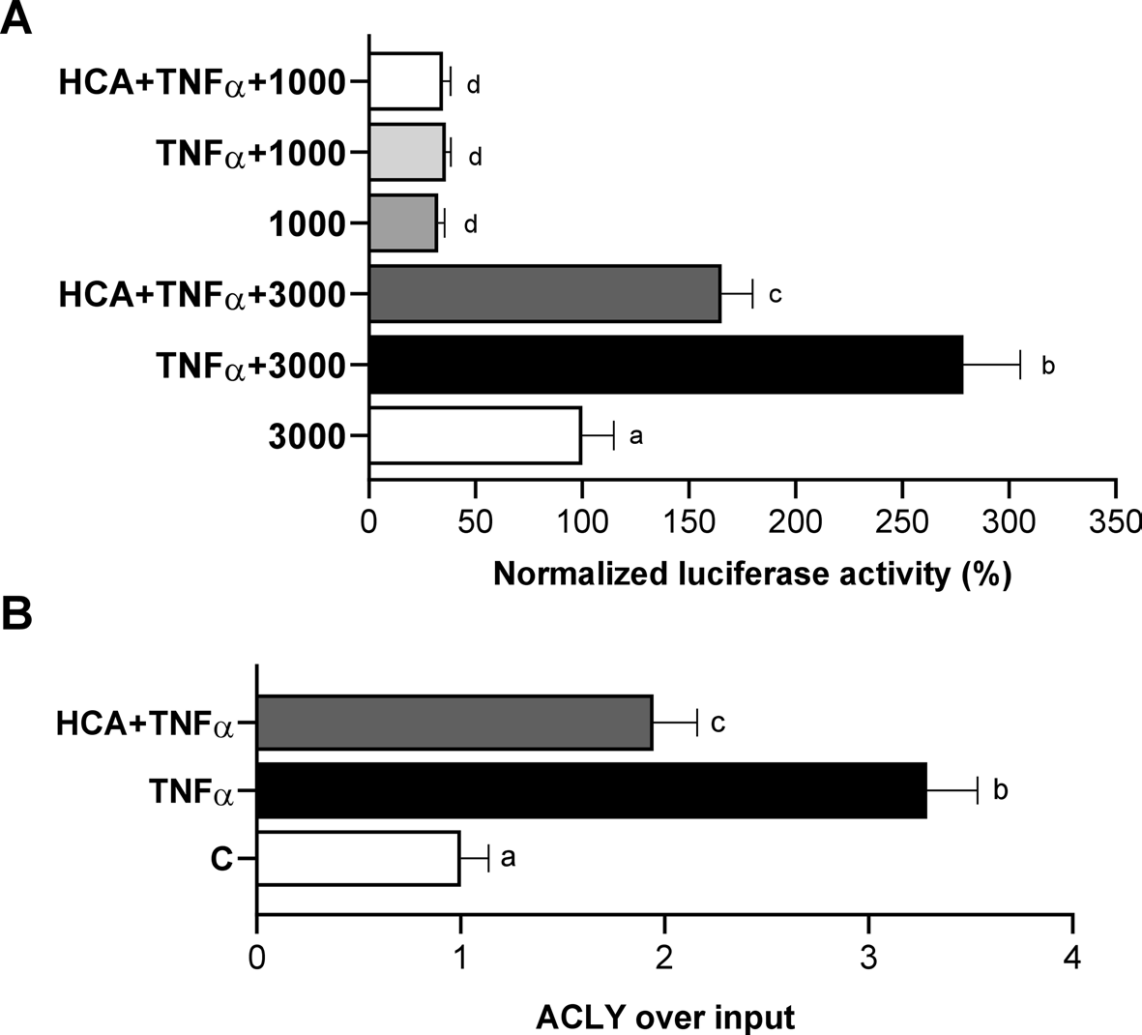
ACLY under NF-kB ACLY-induced transcriptional control in TNFα triggered hepatocytes. **A** HepG2 cells were transiently transfected for 48 h with pGL3 basic-LUC vectors containing the − 3116/ − 20 bp full-length region of the ACLY gene promoter (3000) or a truncated version of this region (1000). Then, cells were triggered by 5 ng/mL TNFα in the absence (TNFα) or in the presence of 500 μM HCA (TNFα + HCA). Unstimulated cells were used as a negative control (3000 or 1000). The luciferase gene reporter activity was assessed after 24 h. Data were analyzed by one-way ANOVA followed by Tukey’s multiple comparison test. Different letters indicate significant differences between treatments at p < 0.05. **B** TNFα triggered HH in the presence or absence of HCA were used to carry out chromatin immunoprecipitation (ChIP) analysis with an antibody against subunit p65 of NF-κB. Data are representative of 3 independent experiments and are presented as means ± SD (error bars). Different letters above the bars indicate significant differences between treatments at *p* < 0.05, according to Tukey’s post hoc test performed after one-way ANOVA

It is well known that TNFα modulates various inflammatory and immune responses through the activation of NF-kB [39]. As previously reported, TNFα treatment upregulates ACLY through NF-kB in macrophages [36]. More recently, we demonstrated that ACLY-mediated p65 acetylation is required for NF-kB full activation and in turn for ACLY transcriptional upregulation in LPS-triggered macrophages [16]. Therefore, we tested if ACLY overexpression was linked to its own activity and depended on NF-kB in TNFα induced hepatocytes. To this aim, liver cells were transiently transfected with 3000 (a pGL3 basic-LUC vector containing the − 3116/− 20 bp region of the ACLY gene promoter including the NF-κB response element localized at − 2048/− 2038 bp) or 1000 (a truncated version without the NF-κB response element) (Fig. 4A). After 48 h, cells were triggered by TNFα in the presence or absence of HCA. Clearly, ACLY gene promoter activity was drastically smaller in cells transfected with 1000 than 3000 due to the lack of NF-κB response element (Fig. 4A). As expected, TNFα increased 2.8-fold luciferase activity in liver cells transfected with 3000 (Fig. 4A). HCA significantly reduced by 40% the ACLY promoter activity in comparison to TNFα activated cells (3000 + TNFα = 278.6 ± 26.7; 3000 + TNFα + HCA = 165.3 ± 14.5, Fig. 4A). IKK 16, an inhibitor of NF-kB signaling, specifically blocks NF-kB activation [40]. In liver cells transfected by 3000 and triggered by TNFα, IKK 16 halved the luciferase activity (Additional file 2: Fig. S4), confirming that ACLY gene upregulation is under NF-kB control. Moreover, ChIP with a specific antibody against p65 revealed a strong binding of NF-κB to the human ACLY gene promoter upon TNFα treatment of HH which was significantly reduced in the presence of HCA (Fig. 4B).

**Fig. 5:**
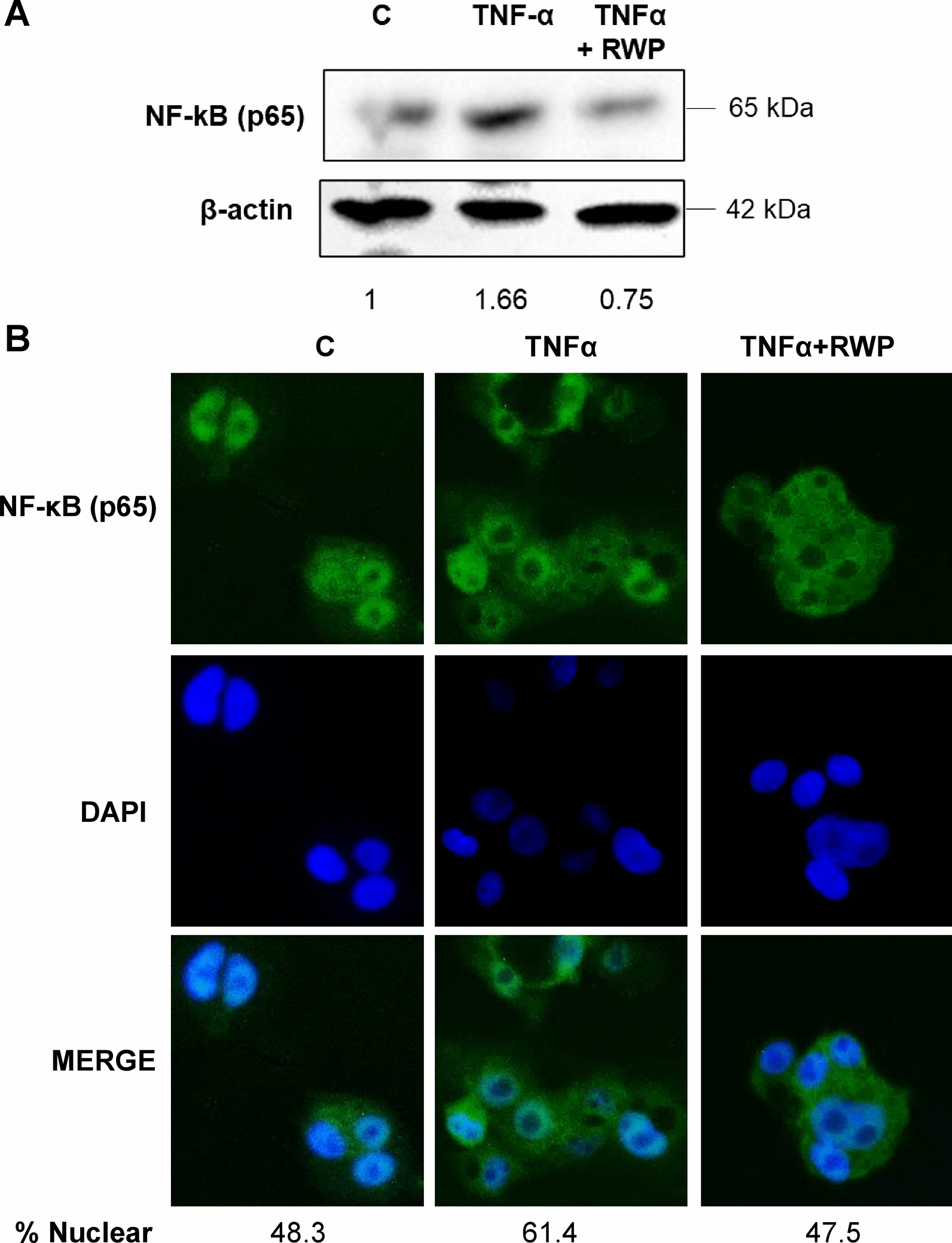
Effect of RWP on NF-kB in TNFα triggered hepatocytes. HH were triggered by 5 ng/mL TNFα in the absence (TNFα) or in the presence of 200 μg/mL RWP (TNFα + RWP). **A** Immunoblots were performed by using specific antibodies against subunit p65 of NF-κB and β-actin. Immunolabeled protein bands were normalized to β-actin by means. Values obtained are reported under western blot images. Protein expression levels in untreated HH (C) were taken as 1. **B** The cellular localization of subunit p65 of NF-kB was identified by immunocytochemistry experiments using a specific antibody. DAPI was used for nuclear staining. Photomicrographs typical of those taken three separate experiments are shown. In **A** and **B**, data are representative of at least 3 independent experiments. % Nuclear = [Total Nuclear Intensity/(Total Cytoplasmic Intensity + Total Nuclear Intensity)] × 100

Next, we analyzed protein expression and cellular localization of NF-kB in TNFα-triggered HH in the presence or in the absence of RWP. We observed a reduction of NF-kB p65 subunit protein levels by slightly more than twofold when RWP was used compared to TNFα-triggered HH (Fig. 5A). After 3 h of treatment with TNFα, the subunit p65 of NF-κB translocated from the cytosol to the nucleus, indeed % Nuclear rose by 26% with respect to unstimulated HH (C, Fig. 5B). Instead, RWP restored % Nuclear to values comparable to C (Fig. 5B).

Our data indicate that ACLY transcription is mediated by NF-κB which in turn requires ACLY activity for its full activation.

### ACLY supports proinflammatory cytokines IL-6 and IL-1β secretion by fostering histone acetylation

**Fig. 6:**
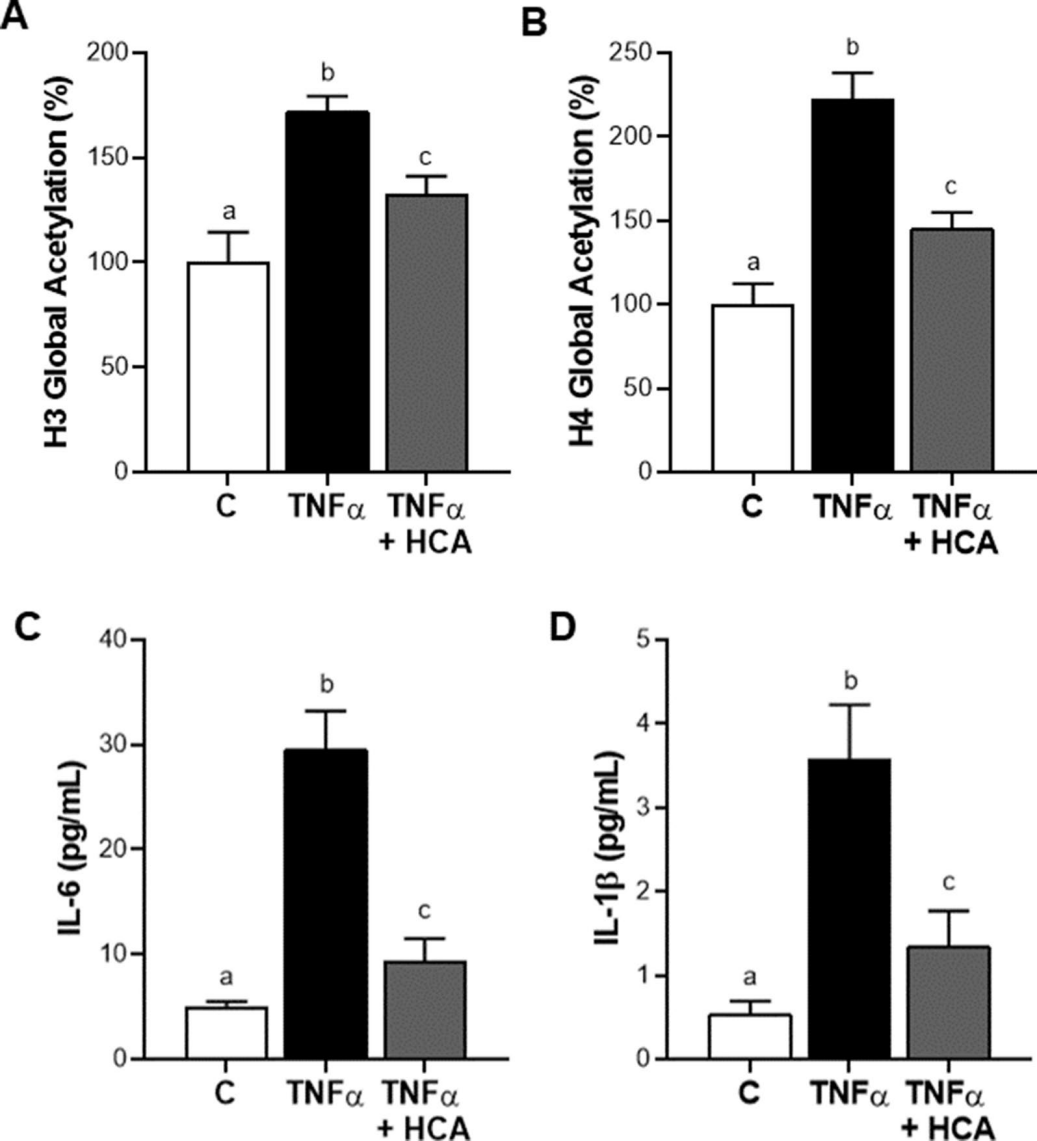
ACLY supports IL-6 and IL-1β secretion by fostering histone acetylation. HH cells were treated with 5 ng/mL TNFα alone (TNFα) or combined with 500 μM HCA (TNFα + HCA). Unstimulated cells (C) were used as negative control. Global acetylation of histone H3 (**A**) and H4 (**B**) was evaluated in different conditions. The concentrations of the proinflammatory cytokines IL-6 (**C**) and IL-1β (**D**) were measured in free-cell culture supernatants following 24 h treatment with TNFα. Values represent the mean values ± SD of three independent experiments with three replicates in each. Statistical analysis was performed by one-way ANOVA followed by Tukey’s multiple comparison test. Different letters indicate significant differences between treatments at p < 0.05

ACLY represents the major source of acetyl-CoA in mammals and is essential for increasing histone acetylation [41] which controls the accessibility to chromatin and gene transcription. Upon TNFα stimulation the levels of global acetylation of both histone H3 and histone H4 significantly increased compared to control HH cells (Fig. 6A, B). Treatment with HCA resulted in significant reductions by 25% in acetylated H3 (Fig. 6A) and by 35% in acetylated H4 (Fig. 6B). Taking into consideration that: (a) ACLY mediates NF-kB full activation and is under the transcriptional control of NF-kB; (b) the activity of transcription factors depends on the state of the chromatin and therefore on its accessibility; (c) NF-kB fosters the transcription of several proinflammatory genes; (d) HCA determines the inhibition of NF-kB and the reduction of histone acetylation, we decided to analyze the levels of proinflammatory cytokines IL-6 and IL-1β, which are both NF-kB target genes. The treatment with TNFα alone resulted in an approximately sixfold increase in both IL-6 (Fig. 6C) and IL-1β (Fig. 6D) secretion. In the presence of HCA, IL-6 levels were decreased by about 30% (Fig. 6C), those of IL-1β by 37% (Fig. 6D) compared to TNFα-activated hepatocytes. Interestingly, RWP, for which we have previously demonstrated a significant effect on gene expression reprogramming through the modulation of the histone acetylation [21], also affected the secretion of both IL-6 and IL-1β. As shown in Additional file 2: Fig. S5, IL-6 (A) as well as IL-1β (B) levels were lowered by about 50% after addition of RWP when compared to HH treated with TNFα alone. These results confirm the central role of ACLY in modulation of proinflammatory genes encoding IL-6 and IL-1β by promoting histone acetylation also in human hepatocytes.

### ACLY and ME1 expression levels in MASH patients

**Fig. 7:**
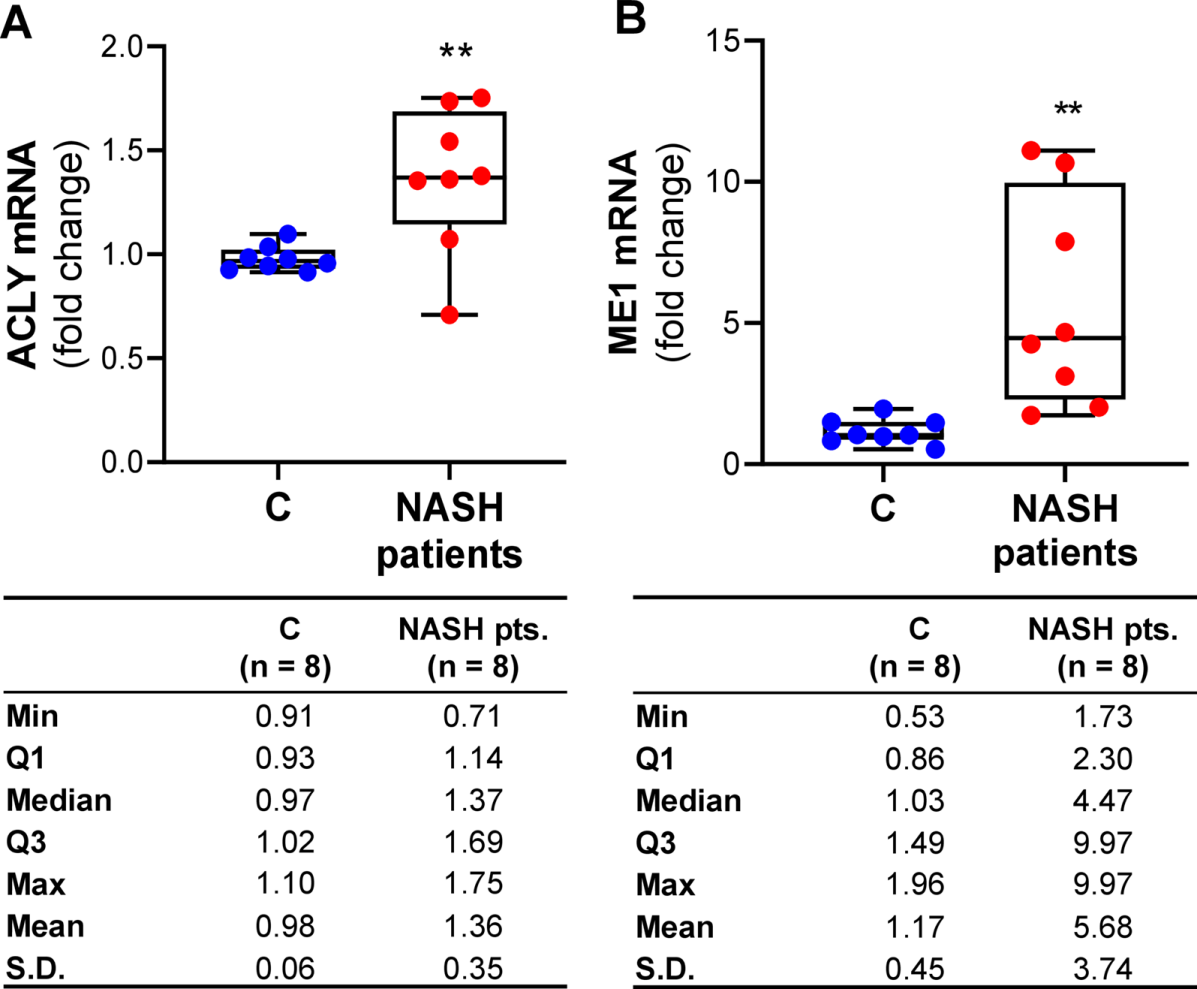
ACLY and ME1 mRNA expression levels in study population. Real time PCR experiments were performed to quantify ACLY (**A**) and ME1 (**B**) gene expression in macrophages from 8 healthy subjects (C) and 8 NASH patients. Data are shown as box plots, where the horizontal line within the boxes is the median, the boxes are the first and third quartiles, and the bars outside the boxes represent the minimum and maximum values. Dots indicate the fold changes of mRNA from each subject enrolled. Differences were significant according to Student’s t-test (**p < 0.01)

In light of the results described above and previously reported about increased levels of ACLY in liver of patients with MASH [20], we investigated whether these dysregulations were also present at a systemic level. Therefore, we evaluated ACLY and ME1 gene expressions in macrophages from MASH patients and healthy controls. ACLY resulted overexpressed in MASH patients: the mean values of ACLY mRNA were ~ 1.4-fold (mean ± SD: 1.36 ± 0.35) greater than in control subjects (mean ± SD: 0.98 ± 0.06) (Fig. 7A). The interquartile range (IQR) in MASH patients, equal to 0.55, suggested that 50% of NASH patients enrolled had ACLY mRNA from 1.14 to 1.69-fold changes of healthy subjects (Fig. 7A). We observed a 4.5-fold increase in ME1 mRNA in MASH patients (mean ± SD: 5.68 ± 3.74, median 4.47) compared to controls (mean ± SD: 1.16 ± 0.45, median: 1.03) (Fig. 7B). Noteworthy, parallel to what happens in hepatocytes, both ACLY and ME1 are overexpressed and ME1 is more markedly expressed than ACLY.

### HCA plus RWP repress ACLY and ME1 and reduce IL-6 and IL-1β proinflammatory cytokines

**Fig. 8:**
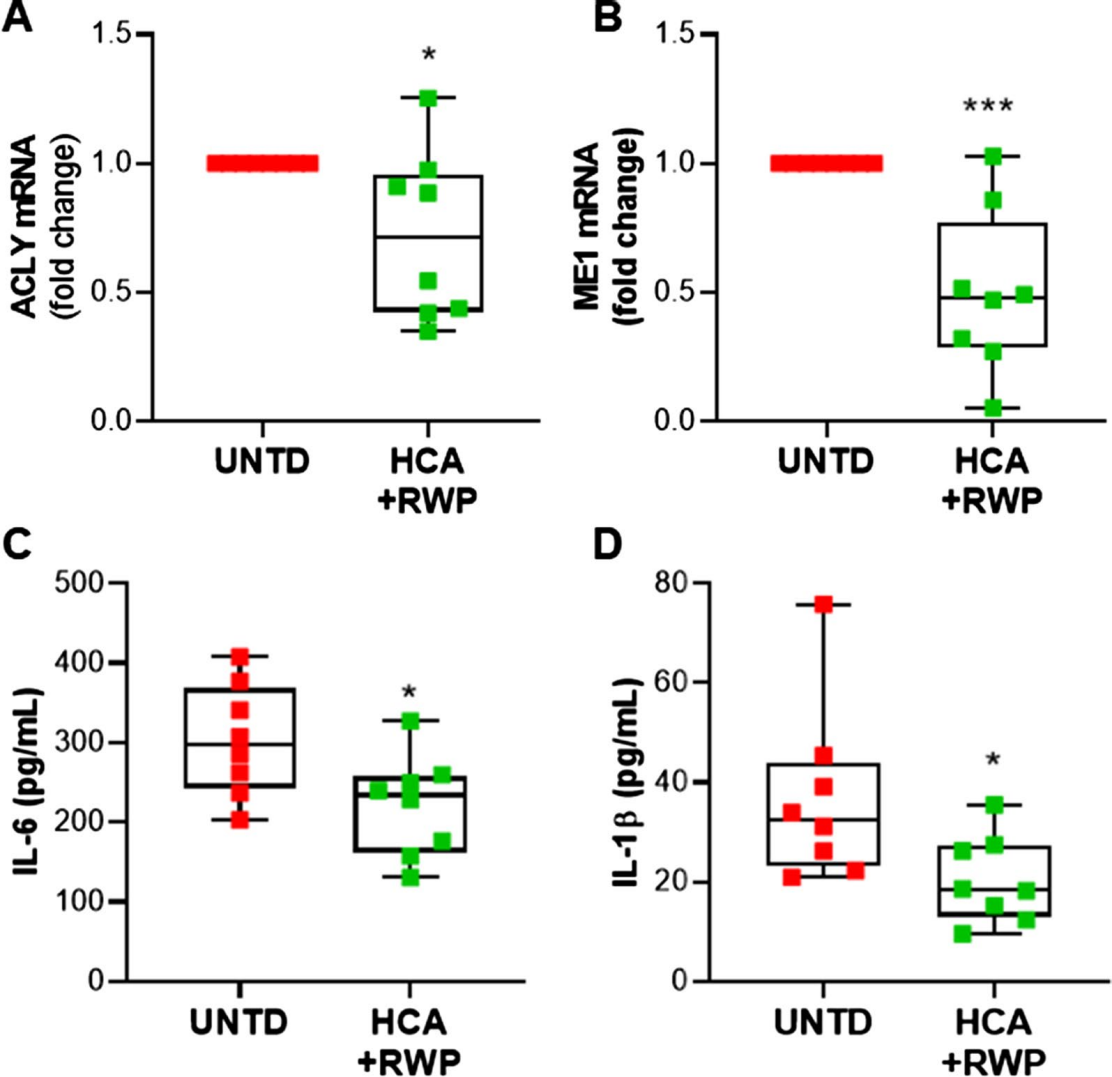
Effects of co-treatment with HCA and RWP in MASH patients. Macrophages from 8 NASH patients were treated with 500 µM HCA + 200 μg/mL RWP for 24 h. Real-time PCR experiments were performed to evaluate ACLY (**A**) and ME1 (**B**) mRNA levels expressed as fold change of unstimulated cells (UNTD). The concentrations of the proinflammatory cytokines IL-6 (**C**) and IL-1β (**D**) were determined in free-cell culture supernatants. Data are shown as box plots, where the horizontal line within the boxes is the median, the boxes are the first and third quartiles, and the bars outside the boxes represent the minimum and maximum values. Dots refer to each subject enrolled. Differences were significant according to Student’s t-test (*p < 0.05 and ***p < 0.001)

Once we confirmed the upregulation of both ME1 and ACLY, we decided to treat macrophages differentiated PBMCs from MASH patients with HCA + RWP to evaluate the effects on both the expression of these genes and specific markers of MASH. In RT-qPCR experiments, for the calculation of ΔΔCt, we subtracted the Ct of the untreated cells (UNTD) from the Ct of UNTD and HCA + RWP treated PBMCs. Consequently, the 2^ΔΔCt^ of UNTD was always equal to 1. As expected, the co-treatment with HCA and RWP significantly (p < 0.05) reduced by ~ 30% ACLY mRNA (mean ± SD: 0.72 ± 0.33) with a range spreading from 0.35 to 1.25-fold changes of control (Fig. 8A). ME1 mRNA content was reduced by even more than half (mean ± SD: 0.43 ± 0.29) when cells were treated with HCA + RWP (Fig. 8B). Except for one patient in whom ME1 was unaffected by HCA + RWP treatment, all the others had ME1 mRNA lower than UNTD (Fig. 8B). Finally, we focused on pro-inflammatory cytokines IL-6 and IL-1β, which represent MASH biomarkers [42, 43]. At the end of 24 h treatment, the cell culture supernatants were collected from UNTD and HCA + RWP cells. We observed significant reductions (p < 0.05) in the secretion of both cytokines following treatment: IL-6 concentration decreased from 302.8 (± 69.9) in UNTD to 221.2 (± 63.4) pg/mL in HCA + RWP treated cells (Fig. 8C); IL-1β levels reduced nearly by the half from 36.9 (± 17.8) in UNTD to 20.4 (± 8.7) pg/mL in HCA + RWP treated cells (Fig. 8D). These results strengthen the role of both ACLY and ME1 in MASH and open glimmers on the possibility of using them as biomarkers as well as drug targets for MASH.

## Discussion

MAFLD development and its progression to MASH remains a serious global issue. Indeed, a high mortality is linked to hepatic injury and chronic inflammation, conditions with a high risk of developing liver failure and a set of cancers including HCC [44, 45]. In recent years, our understanding of MAFLD/MASH and liver cancer has been transforming, as confirmed by the changes from NAFLD to MAFLD and from NASH to MASH which are not just a formal event. As a matter of fact, metabolic dysfunction is a hallmark of both MAFLD and MASH emphasizing as these pathological conditions involve intrahepatic and extrahepatic components [46]. Nowadays, we are better recognizing the critical function of metabolic changes in inflammation, immune cell activation and gene expression reprogramming underlying countless inflammatory diseases. In this scenario, we have investigated the role of ACLY in modulating liver function. For this purpose, we employed primary human hepatocytes in which liver damage was triggered by TNFα. Indeed, it is well known the pivotal role of TNFα in the pathogenesis of MASH. TNFα is early produced during the course of MAFLD with a gradual increase related to the disease severity and with higher levels in MASH than in MAFLD. Not only Kupffer cells but also immune cells infiltrating the liver produce TNFα in MAFLD. Furthermore, a relationship between TNFα secretion and hepatic insulin resistance has been reported, thus linking inflammation and metabolism as possible driver for the MAFLD-related extrahepatic conditions [30, 31, 47].

We have previously shown that a metabolic reprogramming involving ACLY, as part of the citrate pathway, is critical for M1 macrophage activation also induced by TNFα [14]. Since it has been recently demonstrated a key role of ACLY in immunometabolism in activated macrophages [14, 48] while its essential function in the biosynthesis of lipids in the liver has long been known, this immunometabolic enzyme could represent a unique target for MASH due to its multifaceted nature.

Our findings indicate for the first time that ACLY activation along with ME1 upregulation sustain a metabolic and epigenetic reprogramming in TNFα-stimulated hepatocytes.

As a matter of fact, our data beyond demonstrating the impact on fatty acid accumulation, point out ACLY as a driver of gene expression reprogramming by affecting epigenetics in TNFα-triggered HH. Indeed, ACLY-derived acetyl-CoA needed for histone and p65 acetylation to foster the transcription of pro-inflammatory genes among which IL-6 and IL-1β. Moreover, ACLY-dependent early NF-kB full activation in turn upregulated ACLY thus building a positive cycle that was self-sustaining. In this way, ACLY is always kept overexpressed because its function is also required to modulate the redox status in damaged hepatocytes. In physiological condition, ROS are essential to cellular defense, but ROS overproduction is a common trait of many metabolic diseases [49, 50]. Oxidative stress is a major feature of MAFLD and MASH since unbalanced ROS production and lipid peroxidation products were found in patient as well as in animal model of MASH [17, 51, 52]. Hepatic O2^**.**−^ can come from multiple cellular pathways, as well as oxidative phosphorylation, Cytochrome P450 activity, NADPH oxidase activity. Its production opens the doors for the generation of all other reactive oxidants. O2^**.**−^ is converted by superoxide dismutases into hydrogen peroxide (H_2_O_2_), which in turn is the substrate of the Fenton or the Haber- Weiss reaction for the generation of hydroxyl radicals (·OH) [53]. Here we have demonstrated that ACLY inhibition reverted NADPH-dependent ROS and MDA together with GSSG increase induced by TNFα in hepatocytes. Likely**,** in this state NADPH, a product of coupled ACLY and ME1 enzymes, was required for the biosynthesis of fatty acids and pro-inflammatory molecules, such as O2^**.**−^via NADPH oxidase. Therefore, it could be hypothesized that, in this context, NADPH may be less available for the antioxidant defenses of cells, such as glutathione reductase which reduces GSSG in GSH by exacerbating the pro-inflammatory condition. Notably, we also found that RWP from *Aglianico del Vulture* red wine was able to decrease oxidative damage, while the addition of malate, a downstream metabolite of ACLY, abolished this effect. Indeed, OAA-derived malate is synthesized by the cytosolic enzyme malate dehydrogenase 1. Malate was able to suppress the protective effect against oxidative damage exerted by both HCA and RWP. Since malate can be substrate of ME1, thus producing the electron donor NADPH, the combination of malate and NADPH completely reverted ROS lowering induced by HCA or RWP in TNFα-triggered hepatocytes.

Interestingly, both ACLY and ME1 genes were overexpressed in PBMC-derived macrophages from MASH patients, highlighting how this change was not confined only to the liver but was a systemic alteration. Our investigation on macrophages has been suggested by the growing literature pointing out the involvement of these cells in the development of inflammation, steatosis, and fibrosis linked to MASH [54, 55] mainly due to a modulation of their polarization and consequently of their phenotype [56].

In light of these results, we treated PBMC-derived macrophages from MASH patients with HCA, ACLY inhibitor, plus RWP obtaining a diminished secretion of IL-6 and IL-1β pro-inflammatory cytokines along with lowered ACLY and ME1 mRNA levels. Therefore, ACLY activation is critical not only for lipid metabolism but also for oxidative stress, gene expression reprogramming and pro-inflammatory cytokine secretion in liver. Notably, all these processes concur to hepatocellular injury, MASH pathogenesis and development of cirrhosis and HCC [57]. Thus, suppressing ACLY function can be a promising strategy for MASH prevention and therapy.

## Conclusion

In summary, our study describes a multifunctional role of ACLY in liver and its involvement in MASH development. ACLY inhibition, in addition to the reduction of lipid accumulation, leads to a lowering of oxidative stress, modulation of the gene expression and in turn the secretion of pro-inflammatory cytokines thus contributing significantly to hepatocyte homeostasis. Moreover, ACLY upregulation in PBMC-derived macrophages from MASH patients highlights its function in a systemic perspective making it an attractive diagnostic and therapeutic target of MASH.

### Supplementary Information

**Additional file 1: Methods S1.** The file includes the following additional methods: cell proliferation assay and RNA interference.

**Additional file 2: Figure S1.** Effect of HCA and RWP on primary human hepatocyte cell viability. **Figure S2.** ACLY gene silencing reduces oxidative stress in TNFα triggered hepatocytes. **Figure S3.** Effect of RWP on lipid accumulation and oxidative stress in TNFα-triggered human hepatocytes. **Figure S4.** ACLY-dependent NF-kB binding to ACLY gene. **Figure S5.** RWP affected IL-6 and IL-1β pro-inflammatory cytokines secretion in human hepatocytes.

## Data Availability

The data generated during the current study are available from the corresponding author upon request.

## Abbreviations

Ac: Sodium acetate
Acetyl-CoA: Acetyl-Coenzyme A
ACLY: ATP citrate lyase
ChIP: Chromatin immunoprecipitation
CIC: Mitochondrial citrate carrier
GSH: Reduced glutathione
GSSG: Oxidized glutathione
HCA: Hydroxycitrate
HCC: Hepatocellular carcinoma
HH: Primary human hepatocytes
HIF-1a: Hypoxia-inducible factor 1-alpha
HRP: Horseradish peroxidase
IFNγ: Interferon γ
IL-1β: Interleukin-1β
IL-6: Interleukin-6
LPS: Lipopolysaccharide
M: Sodium malate
MAFLD: Metabolic (Dysfunction)-Associated Fatty Liver Disease
MASH: Metabolic (Dysfunction)-Associated Steatohepatitis
MDA: Malondialdehyde
ME1: Malic enzyme 1
NADPH: β-Nicotinamide adenine dinucleotide 2′-phosphate reduced
NAFLD: Non-alcoholic Fatty Liver Disease
NASH: Nonalcoholic steatohepatitis
NF-kB: Nuclear factor Kappa B
NO: Nitric oxide
NP40: Nonidet P40
O2^**.**^^−^: Superoxide anion
OAA: Oxaloacetate
PBMC: Peripheral blood mononuclear cell
PBS: Phosphate buffered saline
PCA: Perchloric acid
ROS: Reactive oxygen species
RWP: Red wine powder
TBA: Thiobarbituric acid
TNFα: Tumor Necrosis Factor-α
