# Supplementary material for: ACLY as a modulator of liver cell functions and its role in Metabolic Dysfunction-Associated Steatohepatitis
Source: J Transl Med. 2023 Aug 24;21:568. doi: 10.1186/s12967-023-04431-w (PMC10463545; doi:10.1186/s12967-023-04431-w)
Supplement: Supplementary file 1 — Additional file 1: Methods S1. The file includes the following additional methods: cell proliferation assay and RNA interference. [file 12967_2023_4431_MOESM1_ESM.docx]

**Additional file 1: Methods S1**

**Cell proliferation assay**

Human Hepatocytes were seeded at a density of 2×10^3^ cells/well in a 96-well plate and allowed to attach overnight. Then, cells were treated with 500 µM HCA or 200 µg/mL RWP. The effects on cells proliferation were evaluated 72 hours later by CellTiter-Glo^®^ 2.0 Cell Viability Assay (Promega, Madison, WI, USA) as per the manufacturer’s instructions. In brief, CellTiter-Glo^®^ 2.0 reagent (100 μL) was added in each well and the plate was shaken for 2 minutes. At the end of 15 min – incubation, luminescence was measured on GloMax^®^ Discover Microplate Reader (Promega).

**RNA interference**

RNA interference experiments were performed for transiently silencing ACLY gene. HH cells were transfected for 48 h with a specific small interfering RNA (siRNA) targeting human ACLY (s915, Validated Silencer^®^ Select, Thermo Fisher Scientific) or control scramble siRNA (4390843, Thermo Fisher Scientific) using Lipofectamine RNAiMax Reagent according to manufacturers’ guidelines.
