# Supplementary material for: ACLY as a modulator of liver cell functions and its role in Metabolic Dysfunction-Associated Steatohepatitis
Source: J Transl Med. 2023 Aug 24;21:568. doi: 10.1186/s12967-023-04431-w (PMC10463545; doi:10.1186/s12967-023-04431-w)
Supplement: Supplementary file 2 — Additional file 2: Figure S1. Effect of HCA and RWP on primary human hepatocyte cell viability. Figure S2. ACLY gene silencing reduces oxidative stress in TNFα triggered hepatocytes. Figure S3. Effect of RWP on lipid accumulation and oxidative stress in TNFα-triggered human hepatocytes. Figure S4. ACLY-dependent NF-kB binding to ACLY gene. Figure S5. RWP affected IL-6 and IL-1β pro-inflammatory cytokines secretion in human hepatocytes. [file 12967_2023_4431_MOESM2_ESM.docx]

**Additional file 2: Additional Figures**


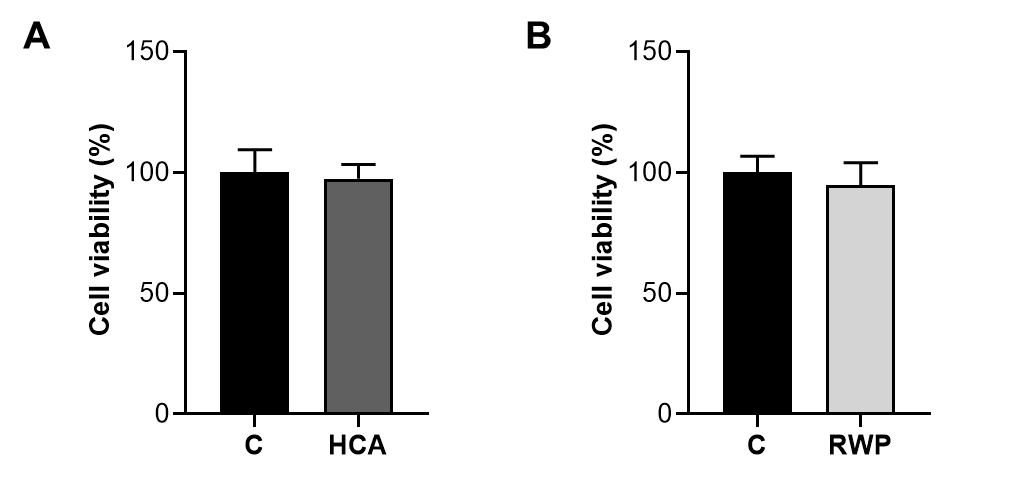


**Figure S1:** **Effect of HCA and RWP on primary human hepatocyte cell viability.** Primary human hepatocytes were treated with 500 µM HCA (**A**) or 200 μg/mL RWP (**B**), and cell viability was assessed after 72 h exposure. The mean values ± SD of three independent experiments with four replicates in each are shown. Differences were not significant according to Student's t-test.


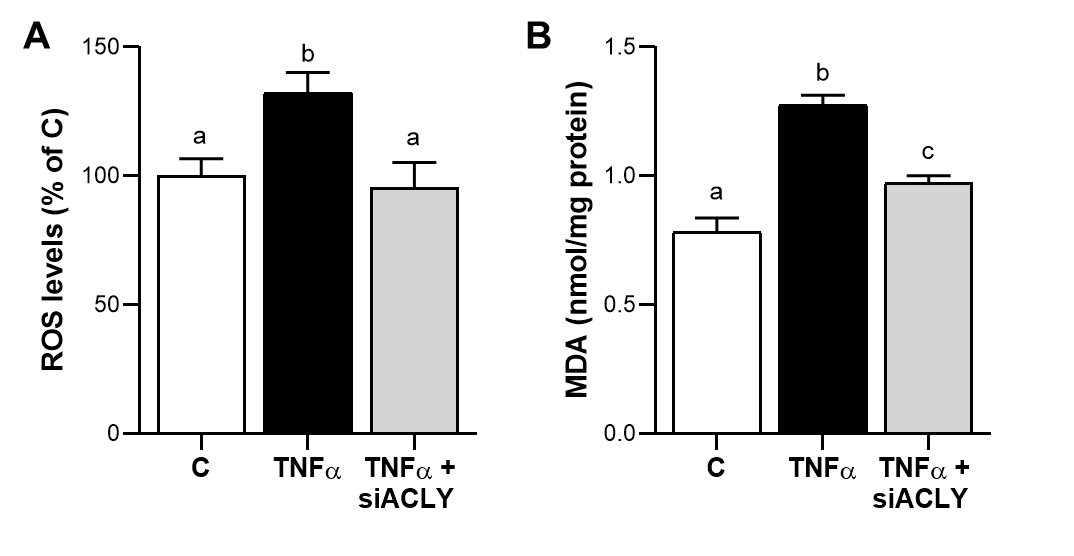


**Figure S2:** **ACLY gene silencing reduces oxidative stress in TNFα triggered hepatocytes.** Primary human hepatocytes were transfected with siRNA targeting human ACLY (siACLY) or control scramble siRNA for 48 h and then treated with 5 ng/mL TNFα. Following 24 h ROS (**A**) and MDA (**B**) levels were measured. In (**A**) values are expressed as the percentage of unstimulated cells (C, set at 100%). Statistical significance of differences was evaluated by using one-way ANOVA followed by Tukey’s multiple comparison test. Different letters indicate significant differences between treatments at p < 0.05.

05.


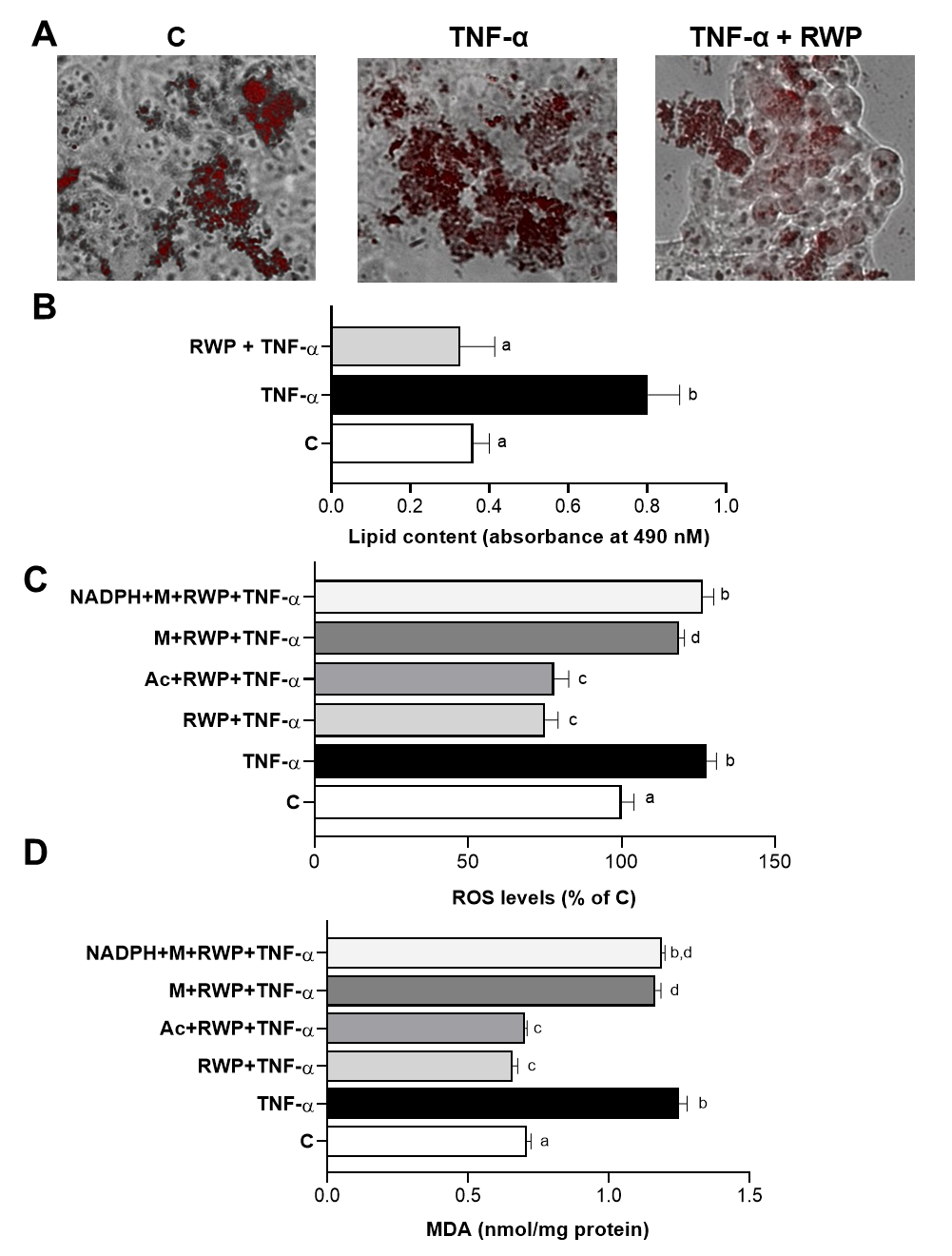


**Figure S3:** **Effect of RWP on lipid accumulation and oxidative stress in TNFα triggered human hepatocytes.** HH cells were triggered by 5 ng/mL TNFα in the absence (TNFα) or in the presence of 200 μg/mL RWP (TNFα + RWP). Unstimulated cells (C) were used as a negative control. (**A**) Representative photomicrographs of intracellular lipids staining from three separate experiments. (**B**) Quantitative assessment of Oil Red O staining. In **(C-D)** TNFα + RWP cells were cotreated with 5 mM sodium acetate (Ac) or 5 mM sodium malate (M) alone or in combination with 500 μM NADPH for 24 h and ROS (**C**) and MDA (**D**) levels were measured. ROS were expressed as the percentage of C, set at 100%. Data are shown as mean ± SD (error bars) and derived from 3 experiments with at least three replicates in each. Statistical significance of difference was evaluated in (**B-D**) by using one-way ANOVA followed by Tukey’s multiple comparison test and different letters indicate significant differences between treatments at p < 0.05.

**Figure S4:** **ACLY-dependent NF-kB binding to ACLY gene.** HepG2 cells were transiently transfected for 48 h with pGL3 basic-LUC vectors containing the −3116/−20 bp full-length region of the ACLY gene promoter (3000) or a truncated version of this region (1000). Then, cells were triggered by 5 ng/mL TNFα in the absence (TNFα) or in the presence of 20 μM IKK inhibitor VII (IKK 16) (TNFα + IKK 16). The luciferase gene reporter activity was assessed after 24 hours. Data were analyzed by one-way ANOVA followed by Tukey’s multiple comparison test. Different letters indicate significant differences between treatments at p < 0.


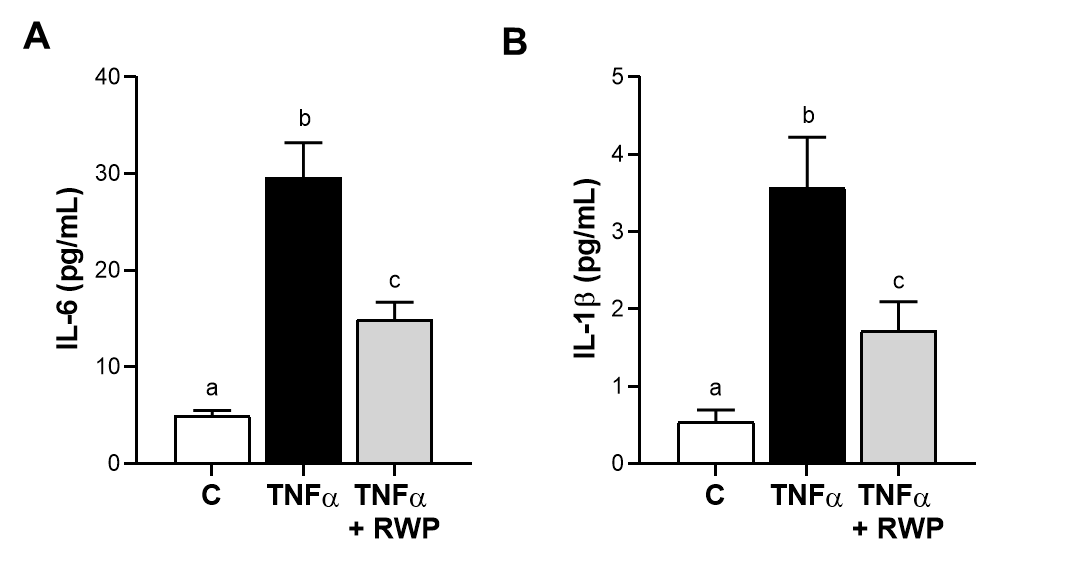


**Figure S5:** **RWP affected IL-6 and IL-1β pro-inflammatory cytokines secretion in human hepatocytes.** HH were treated with 5 ng/mL TNFα alone (TNFα) or combined with 200 μg/mL RWP (TNFα + RWP). Unstimulated cells (C) were used as negative control. The concentrations of the proinflammatory cytokines IL-6 (**A**) and IL-1β (**B**) were measured in free-cell culture supernatants following 24 h treatment with TNFα. Values represent the mean values ± SD of three independent experiments with three replicates in each. Statistical analysis was performed by one-way ANOVA followed by Tukey’s multiple comparison test. Different letters indicate significant differences between treatments at p < 0.05.
